# *Ascaris lumbricoides* infection in captive black sakis (*Chiroptes utahickae*): a case report and implications for zoonotic transmission

**DOI:** 10.29374/2527-2179.bjvm008924

**Published:** 2025-04-08

**Authors:** Bruna Emely Pereira Barbosa, Arthur Carlos da Trindade Alves, Bernardo de Paula Miranda, Beatriz Araújo dos Santos, Agatha Campinho Belsito, Isadora de Fátima Braga Magalhães, Luciano Antunes Barros

**Affiliations:** 1 Facultat de Veterinària, Universitat Autònoma de Barcelona, Bellaterra, Spain.; 2 BioParque do Rio (Zoological Park), Rio de Janeiro, RJ, Brazil.; 3 Instituto de Veterinária, Setor de Medicina de Animais Selvagens, Universidade Federal Rural do Rio de Janeiro, Seropédica, RJ, Brazil.; 4 Self-employed veterinarian, Rio de Janeiro, RJ, Brazil.; 5 Departamento de Saúde Coletiva Veterinária e Saúde Pública, Escola de Veterinária, Universidade Federal Fluminense, Niterói, RJ, Brazil.

**Keywords:** parasitism, primates, zoonosis, parasitismo, primatas, zoonose

## Abstract

*Ascaris lumbricoides* infections represent a major global health challenge, affecting both humans and non-human primates. In non-human primates, *A. lumbricoides* infections pose a significant threat to conservation efforts, as they can impact both captive and wild populations, causing complications ranging from mild to severe. This study reports the case of two black sakis (*Chiroptes utahickae*) kept under human care in a zoo—a male and a female—presented for routine examination due to weight loss. Ultrasonography revealed a substantial intestinal infestation of large roundworms. The female was treated with pyrantel pamoate, and the male with fenbendazole, both with successful outcomes. The worms were sent for morphological and molecular identification, and the animals were submitted for new examinations. This case underscores the importance of preventive veterinary check-ups in zoos, particularly for parasites that pose zoonotic risks, to ensure both animal and human health.

## Resumo

Infecções por *Ascaris lumbricoides* representam um grande desafio para a saúde global, afetando tanto humanos quanto primatas não humanos. Em primatas não humanos, essas infecções representam uma ameaça significativa para os esforços de conservação, pois podem impactar tanto populações cativas quanto selvagens, causando complicações que variam de leves a graves. Este estudo relata o caso de dois cuxiús-pretos (*Chiropotes utahickae*) mantidos sob cuidados humanos em um zoológico—um macho e uma fêmea—apresentados para exame de rotina devido à perda de peso. A ultrassonografia evidenciou uma infestação intestinal substancial por vermes redondos de grande porte. A fêmea foi tratada com pamoato de pirantel e o macho com fenbendazol, ambos com sucesso. Os helmintos foram enviados para identificação morfológica e molecular, e os animais foram submetidos a novos exames. Este caso destaca a importância de check-ups veterinários preventivos em zoológicos, especialmente para parasitas com potencial zoonótico, a fim de garantir a saúde humana e animal.

## Introduction

Ascaridiasis, caused by nematodes of the genus *Ascaris*, is a prevalent parasitic infection that affects both human and non-human primates, particularly in tropical and subtropical regions (Kightlinger et al., 1995; Caraballo & Acevedo, 2011; Mbaya & Udendeye, 2011; Medkour et al., 2020; Rondon Robayo et al., 2022). This parasitic infection, considered as a neglected tropical disease (NTD), is one of the most common and widely distributed helminthic diseases, causing substantial morbidity across diverse geographic regions (Brooker & Pullan, 2013). In humans, *A. lumbricoides* can lead to a range of health problems, including abdominal pain, malnutrition, and intestinal obstruction due to their large size (Akgun, 1996; Villamizar et al., 1996). Similarly, non-human primates, monkeys and apes, can be affected by this parasite (Michaud et al., 2003; Cooper & Redmond, 2016; Stephens et al., 2017; de la Hoz et al., 2020).

*Ascaris lumbricoides* male worms measure between 15 and 31 cm in length; the posterior end is curved ventrally and features a blunt tail. Females range from 20 to 49 cm long; the vulva is located at the anterior end, which corresponds to about one-third of the body length. The developmental cycle is direct (Hall et al., 1992; Taylor et al., 2017). The eggs are highly resistant to extreme temperatures and they can remain viable on the soil for many years ( Hall et al., 1992; Taylor et al., 2017; Nisha et al., 2019). After ingestion, the larvated egg hatches in the small intestine, the L3 larva penetrates the intestinal mucosa, and then travels to the liver. The larva then moves from the bloodstream to the lungs and subsequently to the small intestine, via the bronchi, trachea, and pharynx. In the intestine, it undergoes its final molt and the young adult worms establish themselves in the lumen of the small intestine. Eggs are then shed, restarting the cycle by contaminating new hosts through the ingestion of contaminated water or food. Reinfection depends on constant exposure to *A. lumbricoides* eggs (Hall et al., 1992; Schulz & Kroeger, 1992; Taylor et al., 2017).

Reports indicate that *A. lumbricoides* infections have been documented in zoo environments (Dawet et al., 2013). The proximity of these animals to one another, combined with potential flaws in the sanitation practices, can facilitate the spread of the parasite within these controlled settings (Medkour et al., 2020).

Treatment of *A. lumbricoides* commonly involves the use of anthelmintic medications such as benzimidazole derivatives, pyrantel pamoate, levamisole, and ivermectin. In some communities, plant extracts are also utilized as part of the treatment regimen (Massara & Enk, 2004; Hagel & Giusti, 2010). While these medications are effective in eliminating the parasites from the host's system, their administration must be meticulously managed to ensure complete eradication and to prevent recurrence (Massara & Enk, 2004).

Diagnostic imaging, particularly ultrasound, plays a crucial role in identifying and assessing roundworm infestations (Montórfano, 1999; Wigger et al., 2007; Corda et al., 2022). Ultrasound can provide detailed visualization of the intestinal tract, enabling the accurate detection of parasites and the assessment of infestation severity. This non-invasive technique is essential for guiding treatment decisions and monitoring the effectiveness of therapeutic interventions (Montórfano, 1999; Wigger et al., 2007; Soni et al., 2015; Corda et al., 2022). To date, there are no documented reports of the ultrasound being used to diagnose *A. lumbricoides* parasitism in a non-human primate specimen.

Furthermore, the significance of regular health check-ups extends beyond the zoo animals themselves to include the human caretakers who work in close contact with them (Oh et al., 2002; Souza, 2011; Dawet et al., 2013). The zoonotic transmission of *A. lumbricoides* can pose health risks to those involved in animal care, highlighting the need for comprehensive health surveillance and preventative measures. Ensuring both animal and human health through routine veterinary and medical check-ups is essential in managing and mitigating the impacts of parasitic infections, particularly zoonotic diseases (Deem, 2007; Souza, 2011). In this context, this study aims to report a case of *A. lumbricoides* infection in two black sakis (*Chiroptes utahickae*) and to discuss the diagnostic methods and treatments employed, highlighting its implications as a zoonotic threat.

## Case presentation

**Figure 1:**
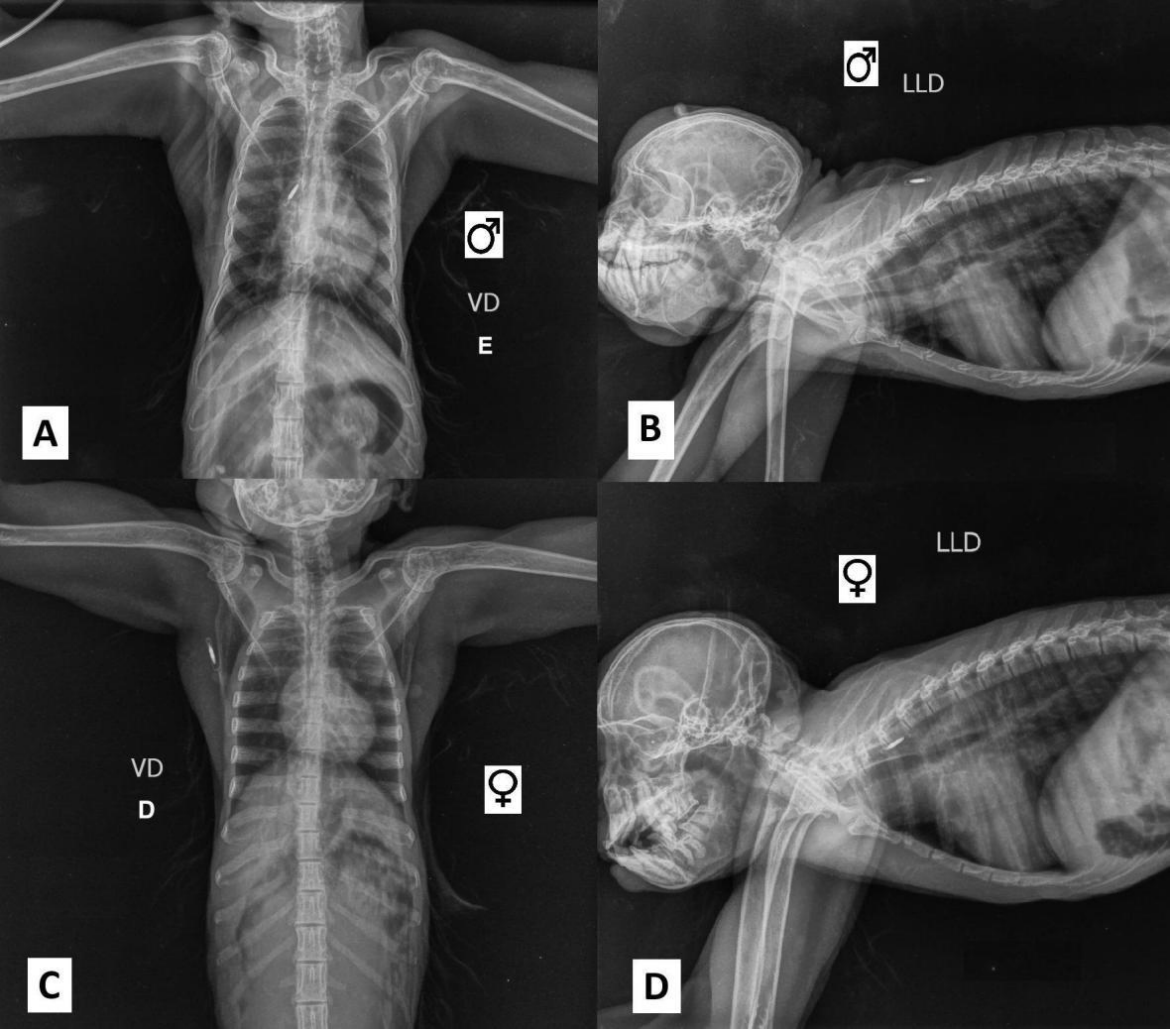
Thoracic radiographs of anesthetized male (A and B) and female (C and D) *Chiroptes utahickae* kept under human care in a zoo showing age-related slight bronchial patterns. Ventrodorsal views (A and C) and lateral views (B and D).

**Figure 2:**
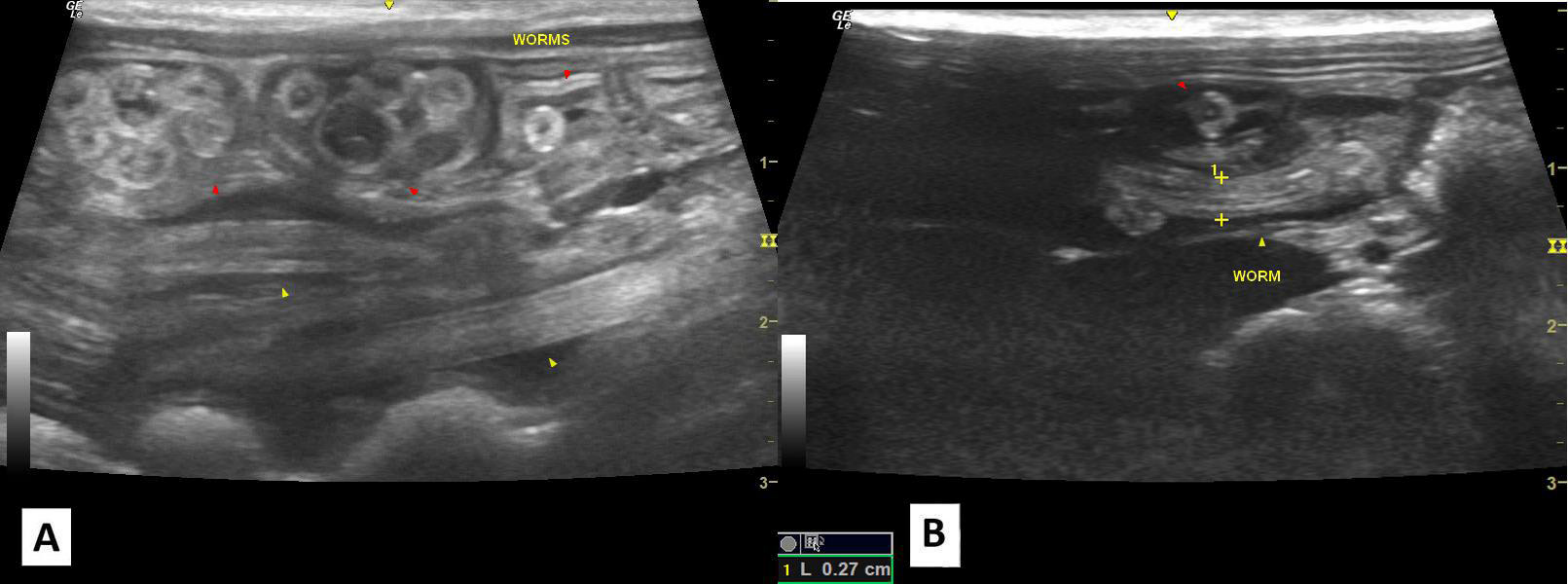
Ultrasonographic images of intestinal nematodes in transverse section (red arrowhead) and longitudinal section (yellow arrowhead) in jejunal segments of two black sakis. Female (A) and male (B).

**Figure 3:**
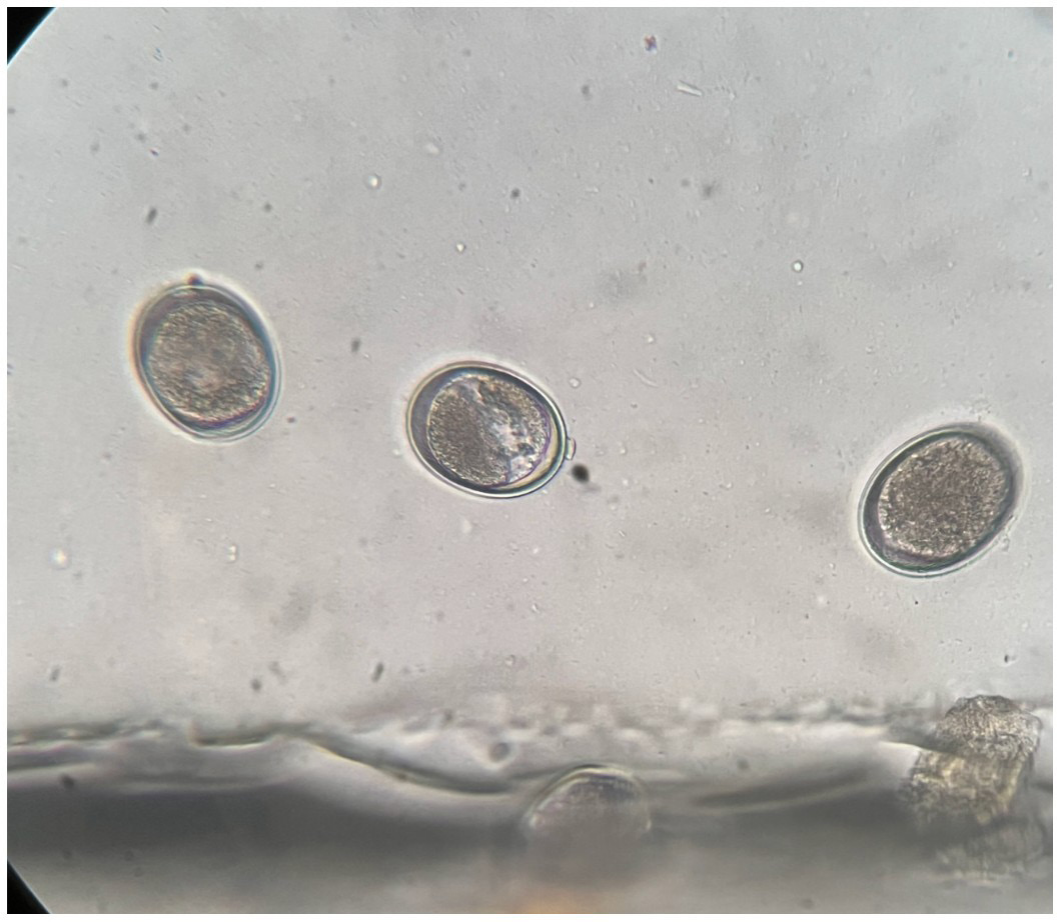
Eggs of *Ascaris lumbricoides* in a fresh *Chiroptes utahickae* fecal sample, coproparasitological examination (Faust technique). Optical microscopy, 40x magnification (+2x digital zoom from a cell phone camera).

Two black sakis (*Chiroptes utahickae*), a male and a female, housed for over 11 years under human care in a zoo in Rio de Janeiro, Brazil, were brought in for a veterinary examination due to reports from their keepers of deterioration in body condition. Their enclosure housed only these two individuals as the sole primate species. Both animals were transported to the veterinary hospital, where they were pre-anesthetized with intramuscular ketamine (7 mg/kg) and midazolam (0.6 mg/kg). Anesthesia was then induced and maintained with isoflurane vaporized through a tracheal tube. A comprehensive evaluation was performed, including physical examination, abdominal ultrasound, thoracic radiographs, echocardiography, blood collection, analysis of fecal samples, and weighing. The male exhibited a poor body condition score of 2/5, while the female’s condition was within the normal range (3/5). Both echocardiograms showed no signs of vascular or cardiac disease, and lung auscultation revealed normal findings in both animals. The x-rays displayed a slight bronchial pattern consistent with normal age-related changes in both patients (Figure 1). The primates exhibited no signs of respiratory disease prior to the examination day. Abdominal ultrasounds were performed using a GE® Logiq E in two-dimensional mode with a high-frequency linear multi frequency transducer ranging from 10 to 22 MHz. The examination revealed intraluminal parasite-like structures which were characterized as elongated structures with 3 to 4 parallel echogenic lines on the longitudinal scan, and in the transverse scan, their appearance resembled a cylindrical “donut.” The duodenal walls were thickened in both patients and had a corrugated appearance, measuring at least 0.25 cm, while the jejunum in both measured between 0.08 cm and 0.15 cm with no wall disturbance. Peristalsis and layer pattern remained unaltered in both animals (Figure 2). Based on the ultrasound findings, deworming was carried out while both the male and female sakis were still under anesthesia. The male was treated with fenbendazole (20 mg/kg), and the female received pyrantel pamoate (10 mg/kg). Both medications were administered in a suspension formulation directly into the stomachs using a lubricated disposable gastric tube (n.12). After administration, the feeding tube was rinsed with 10 ml of water while still in the esophagus of both patients to ensure complete delivery of the medication. Hematological and biochemical analyses were conducted and ​​no noteworthy abnormalities were observed. The coproparasitological examination by sucrose flotation technique (Sheather, 1923) revealed nematode eggs (++), which were oval to round in shape with a thick outer shell (Figure 3).

After recovering smoothly from anesthesia, both animals were kept separately in controlled cages with slotted bottoms to closely monitor parasite elimination, detect any signs of abdominal discomfort, and prevent recontamination, as feces would fall onto clean sheets placed at the bottom of the cages. Simultaneously, their original enclosure was thoroughly cleaned with a fire broom, and all traces of feces were removed.

**Figure 4:**
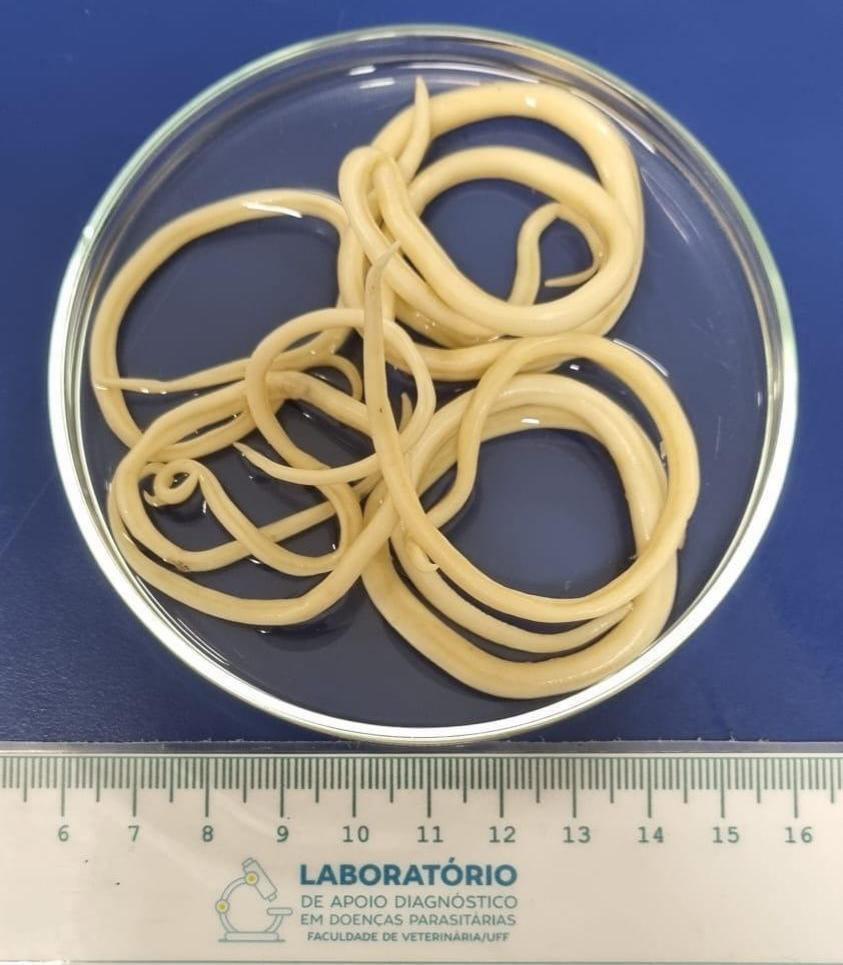
Adults of *Ascaris lumbricoides* preserved in ethanol 70% for morphological identification.

**Figure 5:**
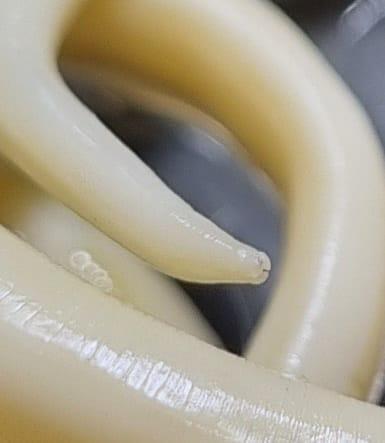
Evidentiation of the trilabial anterior extremity of an adult *Ascaris lumbricoides* specimen.

The female saki expelled four roundworms within 24 hours, ranging in size from 15.5 cm to 21 cm. In contrast, the male saki took three days to eliminate the first seven roundworms and four days to expel the last one. In total, the male saki expelled seven parasites, ranging from 11 cm to 25 cm. The adult worms were preserved in 70% alcohol and sent to the Parasitological Diseases Laboratory of the Universidade Federal Fluminense for morphological identification according to Vicente et al. (1997) (Figure 4 and 5). Subsequently, molecular analysis was performed at the Molecular Epidemiology Laboratory at the same university, confirming *A. lumbricoides* infection.

Using the morphological aspects described by the aforementioned author, the following diagnostic keys were observed for identifying the family: a claviform esophagus without an esophageal bulb, prominent lips, an excretory system shaped like a tuning fork, an excretory pore at the level of the nerve ring, and a non-spiny cuticle. For species suggestion, we observed the absence of cervical alae, a mouth surrounded by three large lips with rows of denticles, no interlabia, and a claviform esophagus without a ventricle. Males had a conical tail without a caudal ala, numerous pre-anal papillae, few post-anal papillae, equal non-winged spicules, and no gubernaculum. Females had a vulva located anterior to the mid-body, a posteriorly directed vagina, and parallel uterine branches.

In order to confirm the diagnostics of *A. lumbricoides*, molecular analysis was performed using conventional polymerase chain reaction (PCR). The sample was preserved in 70% ethanol solution and stored in a refrigerator (2 to 8 °C) until processing. A fragment of the sample, approximately 2 cm, was removed from the ethanol, washed in 1X phosphate-buffered saline (PBS), and ground using a mortar and pestle. The sample was then processed in duplicate and subjected to DNA extraction using the phenol-chloroform method. The DNA quantification in nanograms per microliter was calculated based on the absorbance at 260 nm (A260), and the purity was determined by the A260/230 and A260/A280 ratios. The DNA was stored at -20 °C until use.

**Figure 6:**
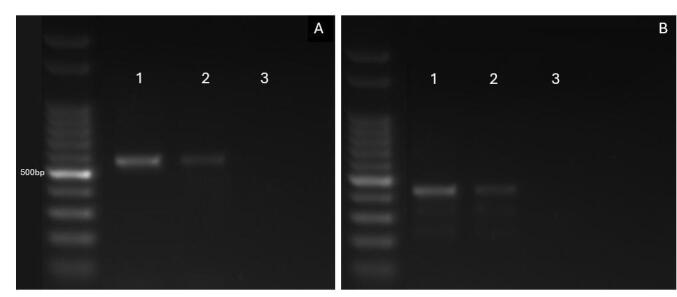
Electrophoresis gel showing PCR results for *Ascaris lumbricoides* detection through the internal transcribed spacer (ITS) region. (A) Lanes 1 and 2 display amplification of the ITS1 region from *Ascaris* samples, with lane 3 being the negative control; (B) Lanes 1 and 2 show amplification of the ITS2 region from *Ascaris* samples, with lane 3 as the negative control. Molecular weight markers (100 bp ladder, Ludwig) indicate the 500-base pair position highlighted in the figure.

The samples were subjected to conventional PCR targeting the internal transcribed spacer (ITS) regions of ribosomal DNA (rDNA) from *Ascaris* spp. following Ishiwata et al. (2004). For the ITS1 region, the forward primer was F2662, 5′-GGCAAAAGTCGTAACAAGGT-3′, and the reverse primer was R3214, 5′-CTGCAATTCGCACTATTTATCG-3′. For the ITS2 region, the forward primer was F3207, 5′-CGAGTATCGATGAAGAACGCAGC-3′, and the reverse primer was R3720, 5′-ATATGCTTAAGTTCAGCGGG-3′. Conventional PCR was performed in a total volume of 25 μL, including PCR buffer (1X, Promega), deoxynucleotide triphosphate solution (dNTPs) (0.2 mM, Ludwig), forward and reverse primers (0.2 μM, Thermofisher), Taq DNA polymerase (0.5 U/µL, Promega), PCR-grade water, and template DNA (600 ng) or water (negative control). The thermocycler reactions (Bio-Rad) were programmed as follows: initial incubation incubation at 95°C for five minutes, followed by 35 cycles of 1 minute at 94 °C, 1 minute at 60 °C, and 1 minute at 72 °C, with a final extension period at 72°C for 10 minutes. PCR products were analyzed via 1.5% agarose gel electrophoresis stained with ethidium bromide and visualized under UV transillumination (Figure 6). Amplicons were purified using the ReliaPrep DNA Clean-up and Concentration System (Promega) and sequenced using the same primers as in the amplification reactions. The nucleotide sequences obtained were analyzed using BioEdit software (Ibis Biosciences) and compared with sequences deposited in the NCBI database for *Ascaris* spp. The DNA extracted from the *Ascaris* specimen yielded concentrations of 137.8 ng/μL and 197.8 ng/μL. The purity ratios were 1.738 and 1.893 for A260/230 and 1.708 and 1.749 for A260/A280. PCR amplification of the ITS-1 and ITS-2 regions produced fragments of 580 bp and 456 bp, respectively, confirming the sample’s identification as *Ascaris* spp. (Figure 6).

A phylogenetic tree was constructed using the MEGA X software with the Maximum Likelihood method and Kimura’s two-parameter model (Kimura, 1980). The bootstrap consensus tree inferred from 1,000 replicates was used to determine the phylogeny of the analyzed taxa, and branches corresponding to partitions reproduced in less than 50% of the bootstrap replicates were collapsed.

**Figure 7:**
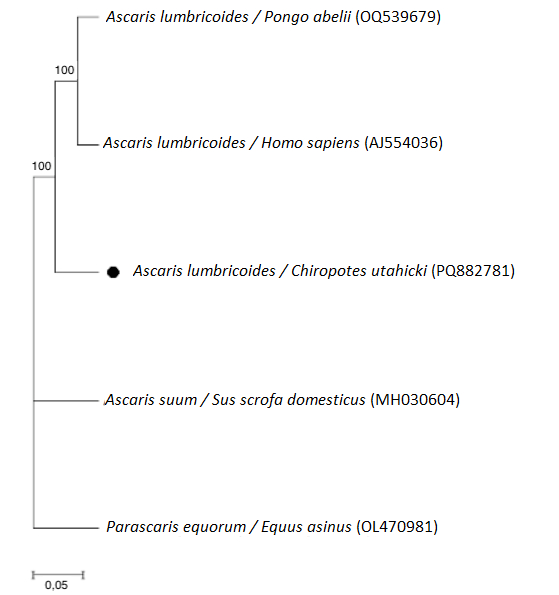
Phylogenetic tree of *Ascaris* spp. using the partial sequence of the ITS-1 gene. All accession numbers correspond to different isolates, followed by their host and GenBank database accession number. The newly generated sequence from this study is marked with a solid black circle.

**Figure 8:**
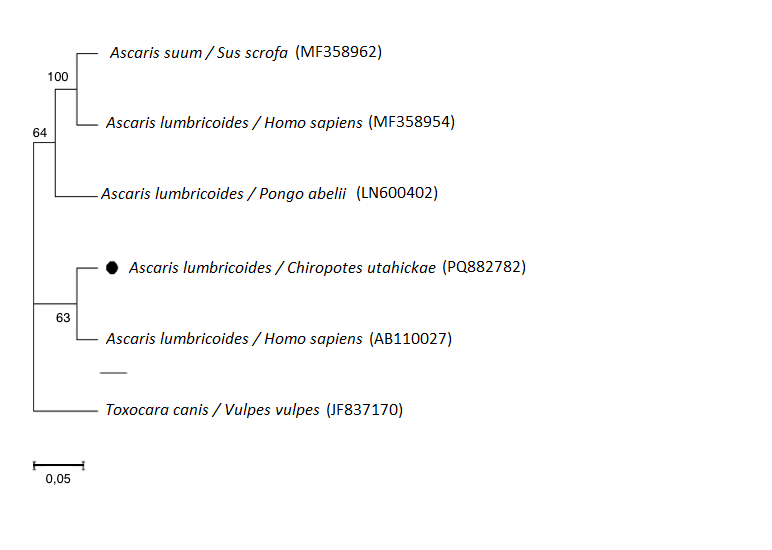
Phylogenetic tree of *Ascaris* spp. using the partial sequence of the ITS-2 gene. All accession numbers correspond to different isolates, followed by their host and GenBank database accession number. The newly generated sequence from this study is marked with a solid black circle.

In the ITS-1 region analysis (Figure 7), the sequence (GenBank accession number PQ882781) formed a highly supported clade (bootstrap = 100) with *A. lumbricoides* sequences isolated from *Homo sapiens* and *Pongo abelii*. Similarly, in the ITS-2 region (Figure 8), the sample from this study (GenBank accession number PQ882782) was also grouped with an *A. lumbricoides* sequence.

As for the host primates, anesthesia and deworming were repeated according to the same protocol after 21 days for both animals. Subsequent coproparasitological examinations showed no eggs, and ultrasound scans revealed no remaining roundworms. Given these results, both animals were returned to their original enclosure. Throughout their stay in the hospital, neither animal exhibited signs of hyporexia, diarrhea, or apathy.

## Discussion

Lilly et al. (2002) performed faecal examinations on indigenous tribes, active researchers and wild local Apes in the Central African Republic. Their study revealed a high prevalence of zoonotic nematodes, suggesting that due to inadequate hygiene practices within these communities, cross-infection between humans and non-human primates could occur. Similarly, Medkour et al. (2020) found homologue findings in the Republic of Congo and Senegal.

This suggests that the geographical proximity between non-human primates (NHPs) and humans can serve as a risk factor for the transmission of parasitic diseases, specially under favorable conditions, such as the weather (tropical or subtropical), high humidity and the deficiency of sanitary conditions (Tshikuka et al., 1995; Walker et al., 2011; Yang et al., 2018). It is important to point out that the zoo that is being discussed in this study is located adjacent to a low-income community with many informal housing, where a significant portion of residents rely on rainwater harvesting tanks as their main source. Additionally, Rio de Janeiro has a tropical climate with frequent rainfall. Given that the sakis’ enclosure is an outdoor space, the soil remains consistently humid, creating an environment conducive to the viability of *Ascaris* spp. eggs (Brooker & Michael, 2000; Lilly et al., 2002).

Considering the unexpected diagnosis of the NHP of this study, it is important to note that the ultrasound played a crucial role in the initial suspicion of ascaridiasis and in assessing the clinical condition of both patients. It guided the veterinarians in the decision to administer antiparasitic treatment with drugs capable of addressing the disease while the animals were still under anesthesia, ensuring a more favorable outcome and full intake of the medication, as it was administered directly into their stomachs. Moreover, this approach allowed for a more comprehensive assessment of the parasite's impact on the hosts, serving as a valuable complement to coproparasitological examination. In our case, the coproparasitological examination was performed after the ultrasound confirmed the presence of parasites, demonstrating the essential complementarity of both diagnostic methods in detection of the disease. In humans, the ultrasound has been used as an important diagnostic tool in ascaridiasis cases, also aiding in the evaluation of other organs that may be affected by the larvae migration, such as the biliary tract and liver (Price & Leung, 1988; Montórfano, 1998; Wu, 2009; Soni et al., 2015). To the best of the authors’ knowledge, there have been no previous report of this diagnostic tool being used to detect ascaridiasis in a neotropical NHP.

There is an ongoing debate in the literature regarding whether *A. lumbricoides* and *Ascaris suum,* a nematode that primarily infect pigs, represent distinct species or variants of the same species (Leles et al., 2012; Zhou et al., 2020). This discussion stems from the significant genetic similarities shared between the two, as well as their morphological overlap. While traditionally classified based on host specificity, with *A. lumbricoides* infecting primates and *A. suum* primarily associated with pigs, molecular evidence suggests frequent gene flow between populations, indicating that cross transmission can occur, thus blurring the lines of differentiation (Leles et al., 2012; Alves et al., 2016; Zhou et al., 2020).

Thus, the molecular analysis of the helminths in this case is of critical importance. Due to the substantial morphological similarities between *A. lumbricoides* and *A. suum*, relying solely on morphological characteristics can lead to misidentification. Traditional morphological methods are essential for approximating species-level identification; however, they lack the precision necessary to differentiate these two closely related species, given their minimal differences (Maung, 1973; Bacelar et al., 2023). Performing molecular analysis, such as PCR targeting specific genomic regions, provides species confirmation and also plays a crucial role in improving our understanding of host-parasite relationships (Zhou et al., 2020).

The sample from this study (GenBank accession number PQ882781) was identified as *A. lumbricoides* through analysis of the ITS-1 region, reinforcing the utility of this region for species identification within the *Ascaris* genus, as previously reported by Sadaow et al. (2018). This approach can be applied to cases involving non-human primates, such as *C. utahickae*. Meanwhile, the analysis of the ITS-2 region emphasized the genetic proximity to *A. lumbricoides* and the formation of a closely related cluster with *A. suum*, which has been previously reported in other studies due to the genetic similarity between these species.

The treatment for both cases of ascaridiasis was effective with both fenbendazole and pyrantel pamoate, although the time required to start eliminating parasites varied between individuals. This difference in response could be attributed to the different parasitic loads between the two sakis observed on the ultrasounds, but it is more likely due to the distinct mechanisms of the medications used. Fenbendazole, a broad-spectrum benzimidazole, disrupts microtubule formation in nematodes, leading to their death. It typically begins to show effects within a few days to a week (Fennell et al., 2008; Taylor et al., 2017). In contrast, pyrantel pamoate causes paralysis of the worms by affecting the neuromuscular junction, which leads to their expulsion from the host’s body through normal peristaltic movements. Pyrantel pamoate generally acts more quickly to kill parasites (Massara & Enk, 2004; Taylor et al., 2017), which was consistent with our observations.

Regarding treatment, it is crucial to note that treating the infected animals alone will not prevent reinfection, as *Ascaris* eggs are highly resistant in the soil and can lead to recurring infections (Hlaing et al., 1987; Henry, 1988; Hall et al., 1992). Therefore, in addition to administering chemical treatment to the affected animals, environmental management and ensuring the quality of water and food is essential. This includes implementing proper soil drainage, providing clean water and food, removing old feces from the enclosure, and conducting regular stool testing for both the zoo staff and the animals. Such measures are vital for maintaining a parasite-free environment (Pérez Cordón et al., 2008; Panayotova-Pencheva, 2013; Bais et al., 2017; Moreno Mañas et al., 2019; Martelli & Krishnasamy, 2023). This comprehensive approach not only enhances the well-being of the animals but also protects the health of the zoo staff and the surrounding community, creating a safer and healthier environment for all. It aligns with the One Health approach and contributes to wildlife conservation efforts.

## Conclusion

In conclusion, the importance of conducting multiple complementary diagnostic tests cannot be overstated when addressing parasitic diseases. In the case of the two black sakis (*C. utahickae*) infected with *A. lumbricoides*, ultrasound proved to be an invaluable tool for accurately assessing the extent of the infection and guiding treatment decisions. Additionally, this case reports the successful treatment of both cases with fenbendazole and pyrantel pamoate, with a slight difference in the time required for elimination of adult parasites. Finally, considering the importance of an One Health approach for wildlife veterinarians, this paper underscores the importance of vigilant monitoring and effective management of parasitic infections in zoo environments.
